# Four new loci associations discovered by pathway-based and network analyses of the genome-wide variability profile of Hirschsprung’s disease

**DOI:** 10.1186/1750-1172-7-103

**Published:** 2012-12-28

**Authors:** Raquel Ma Fernández, Marta Bleda, Rocío Núñez-Torres, Ignacio Medina, Berta Luzón-Toro, Luz García-Alonso, Ana Torroglosa, Martina Marbà, Ma Valle Enguix-Riego, David Montaner, Guillermo Antiñolo, Joaquín Dopazo, Salud Borrego

**Affiliations:** 1 Department of Genetics, Reproduction and Fetal Medicine, Institute of Biomedicine of Seville (IBIS, University Hospital Virgen del Rocío/CSIC/University of Seville, Seville, Spain; 2 Centre for Biomedical Network Research on Rare Diseases (CIBERER), Barcelona, Spain; 3 Department of Bioinformatics, Research Centre Príncipe Felipe, Valencia, Spain; 4 Functional Genomics Node (INB), Research Centre Príncipe Felipe, Valencia, Spain

**Keywords:** HSCR, Pathway-based analysis, Network analysis, GWAS

## Abstract

Finding gene associations in rare diseases is frequently hampered by the reduced numbers of patients accessible. Conventional gene-based association tests rely on the availability of large cohorts, which constitutes a serious limitation for its application in this scenario. To overcome this problem we have used here a combined strategy in which a pathway-based analysis (PBA) has been initially conducted to prioritize candidate genes in a Spanish cohort of 53 trios of short-segment Hirschsprung’s disease. Candidate genes have been further validated in an independent population of 106 trios. The study revealed a strong association of 11 gene ontology (GO) modules related to signal transduction and its regulation, enteric nervous system (ENS) formation and other HSCR-related processes. Among the preselected candidates, a total of 4 loci, *RASGEF1A*, *IQGAP2*, *DLC1* and *CHRNA7*, related to signal transduction and migration processes, were found to be significantly associated to HSCR. Network analysis also confirms their involvement in the network of already known disease genes. This approach, based on the study of functionally-related gene sets, requires of lower sample sizes and opens new opportunities for the study of rare diseases.

## Background

Understanding complex genetic diseases is one of the great challenges for XXI^st^ century medicine. In the past few years, the development of new tools, such as Genome-Wide Association Studies (GWAS) and Next Generation Sequencing (NGS), has allowed the identification of hundreds of genetic variants associated to different diseases that provide new insights of their genetic architecture. Hirschsprung’s disease (HSCR, OMIM 142623) is a nice example of the current situation of many of the complex genetic diseases. HSCR or aganglionic megacolon is a rare developmental disorder of variable penetrance and expressivity, male predominance, and an incidence of 1/5000 newborn human infants. It is a neurocrystopathy characterized by the absence of intramural ganglion cells in the myenteric and submucosal plexuses along a variable portion of the distal intestine. The most widely accepted etiopathogenetic hypothesis for HSCR is based on a defect of craniocaudal migration of neuroblasts originating from the neural crest that, under normal circumstances, reach the small intestine in the 7th week of gestation and the rectum in the 12th week [1].

The HSCR clinical forms with variable extension of the aganglionic segment could be interpreted as interruptions of the migration process in different gestational periods: the earlier the migration arrest, the longer the distal aganglionic intestinal portion. Based on the length of the aganglionic region, the disorder is classified into short segment (S-HSCR: aganglionosis up to the upper sigmoid colon, 80% of cases), long-segment (L-HSCR: aganglionosis up to the splenic flexure and beyond, 17% of cases) and total colonic aganglionosis forms (TCA, 3% of cases). Significantly, HSCR displays a highly variable phenotype with variation in recurrence risk by gender, familiarity, segment length of aganglionosis and associated phenotypes [2]. The reason for most of this variation is largely unknown, although gene discovery has clarified some genotype-phenotype correlations. Numerous molecular genetic studies have identified rare coding mutations in many genes (*RET*, *GDNF, NRTN, PSPN, GFRA1, EDNRB, EDN3, ECE1, NTF3, NTRK3, SOX10, PHOX2B, L1CAM, ZFHX1B, KIAA1279, TCF4, PROK1, PROKR1, PROKR2, NRG1, SEMA3A* and *SEMA3D*) related to HSCR [1-8]. Moreover, several additional HSCR-associated regions, such as 9q31 [9], 3p21 [10,11], 19q12 [10], 16q23 [12], 21q21 [13] or 4q31.3-q32.3 [14] have been described, although the genes underlying such associations have not been identified yet. However, cumulatively, the conventional mutations related to HSCR reported so far explain around 5% of cases, being the vast majority of them L-HSCR/TCA and syndromic forms of the disease [1,2]. Additional phenotypic variation is explained by common low-penetrance polymorphic variants at *RET* (10q11.21) [2,15,16] and *NRG1*[17], but the vast majority of HSCR heritability is still unknown. In other words, all these genes and loci seem to contribute to HSCR phenotype, but associations change according to the population studied and the way of contribution of the different elements is still unclear.

Conventional GWAS, based on single markers, require of large cohorts. This represents an especially remarkable limitation for the case of rare diseases, where typically a limited number of patients are available. In an attempt to overcome this limitation, pathway-based analysis (PBA) [18] has been applied to select groups of functionally-related genes collectively associated to the disease (through their corresponding SNPs) in a first analysis step. Actually, PBA has already been successfully applied to the study of several diseases [19-21]. The application of PBA tests produced a reduced list of candidate genes which was further validated with an independent set of SNPs in a larger population of 106 trios. The result of this validation was the identification of four new loci associated to the disease, *RASGEF1A*, *IQGAP2*, *DLC1* and *CHRNA7*, accounting for different defects in biological processes related to HSCR, such as signal transduction (particularly the Ras pathway) and ENS formation. In addition, network analysis was used as supplementary evidence to support the results found. Network analysis, exploits the information contained in the interactome with the idea that proteins close in the interaction network will have a higher probability of causing the same disease and constitutes a powerful technique to detect gene-disease associations [22-24]. Network analysis has been successfully applied to discover genes in different diseases, such as ataxias [25], Huntington disease [26], schizophrenia [27] or Alzheimer’s disease [28].

## Material and methods

### Subjects

We have included in this study a total of 159 sporadic isolated HSCR trios, composed by the affected patients (127 males, 32 females) and their unaffected parents, all of them originating from Spain. A total of 103 out of the 159 patients were S-HSCR, 31 presented with L-HSCR and the length of aganglionosis was not available for the remaining 25 cases.

For the first part of this study, consisting in the genome wide genotyping study, we selected 53 S-HSCR trios. For further validation studies of the candidate genes resulting from GWAS, we genotyped an independent selection of SNPs in an independent series of 106 trios.

An informed consent was obtained from all the participants for clinical and molecular genetic studies. The study conformed to the tenets of the declaration of Helsinki as well as the requirements established by our Institutional Review Board.

### Genome-wide genotyping

Genome wide genotyping was carried out using the Affymetrix 500 k chip (composed of the 250 k Nsp and the 250 k Sty chips) and the Genechip® System (Affymetrix). Quality controls were as follows: SNPs missing in more than 20% of the samples in the calling process were discarded. SNPs with MAF < 0.5%, with Mendelian errors or not in Hardy-Weinberg equilibrium (in unaffected samples; p-value < 10^-5^) were also discarded. Samples with more than 5% of the SNPs missing were not considered for further analysis. Data are available in the Gene Expression Omnibus (GEO) database (http://ncbi.nlm.nih.gov/geo/) under the identifier GSE33732.

### Conventional gene association analysis in trios

Transmission Disequilibrium Test (TDT) association statistics was carried out as implemented in the PLINK [29] software for the 53 trios included in the genome wide genotyping.

### Pathway-based analysis

The SNPs were ranked according to their p-values obtained in the TDT test and a PBA test [30], as implemented in the GESBAP [31] module of the Babelomics software [32,33], was conducted. Briefly, PBA tests make only use of SNPs mapping within genes or in the neighborhood (here defined as 500 bps up-and downstream of the gene limits). When multiple SNPs map onto the same gene, the SNP with lowest (most significant) p-value is retained. In this way, a list of genes ranked by the p-values of the SNPs mapping onto them is constructed. Then the PBA test analyzes the distribution of sets of functionally related genes across the list. Here we use Gene Ontology (GO) [34] terms to define the functional modules tested. GO terms significantly associated to low p-values are found upon the application of a logistic regression. To avoid false positives due to multiple-testing effects, only GO terms with a FDR-adjusted [35] p-value < 0.05 are declared significant.

### PBA-based two stage study

A widely used strategy involves the genotyping of a number of samples for a large number of markers on a first stage (discovery step), followed by a second genotyping step of a smaller selection of markers in another (larger if possible) subset of samples (validation step). The standard approach considers stage 2 as a replication study and focus on markers statistically significant when stage 2 is considered alone [36].

Here, instead of using a conventional approach based on single marker associations for the discovery step, we have followed a conceptually different approach. Our approach is inspired in an idea that is gaining popularity, that a disease is rarely the consequence of a defect in a single gene, but rather reflects the perturbations of the complex network of functionally related gene modules [23,37,38]. Therefore, we have used PBA to discover gene modules (represented by GO terms) significantly associated to the disease. Then, we consider that genes belonging to such GO modules define a subset of candidate genes to be tested in the second stage. A similar idea has already successfully been used for gene expression analysis [39].

According to the PBA carried out in the discovery stage, a total of 68 candidate genes belonging to the GO modules most associated to the disease were selected because of their lower p-values. A total of 190 new SNPs mapping onto the 68 candidate genes (an average of 1–3 SNP/gene) were subsequently selected for further evaluation. Selection of SNPs within those genes followed 4 main criteria: they were not included in the chip used for the first genotyping study, they should be homogeneously distributed along the entire sequence of the gene (including regions susceptible of being regulatory elements), their reported allelic frequency should be of at least 5% in Caucasian population and should have been identified as Tag SNPs by the HapMap Project. The software PupaSuite [40] was used to help in this section. Large-scale validation genotyping of each SNP was performed in the independent series of 106 trios by Taqman technology using 7900HT Fast Real-Time PCR System (Applied Biosystems, Foster City, California, USA) as previously described. As usual, significance in the transmission of the alleles in the families is tested by an *X*^2^ test, applying a Yates correction (SPSS v.17.0).

One limitation of this approach is that SNPs not mapping within (or close) to genomic elements with a functional annotation will be missed. However, the power for detection of associations of SNPs related to genes with some known function, interaction, etc., is greatly increased by this approach.

### Expression analyses

Human postnatal gut specimens were obtained from endoscopic gut samples biopsied from patients in the University Hospital Virgen del Rocío because of different medical indications. Except for the case of RASGEF1A, whose expression had already been verified in ENS [16], these samples were used to verify gene expression of the genes that resulted associated to HSCR, by both RT-PCR and immunohistochemistry analyses.

The cDNA specimens from those tissue samples were obtained with the gentleMACS™ Dissociator and thermoMACS™ Separator systems (Miltenyi Biotech) following the protocols recommended by the manufacturers. RT-PCR analyses were performed on such cDNA samples to test expression of each of the associated genes. As positive controls we used Brain Human Normal cDNA, Kidney Human Normal cDNA and Liver Human Normal cDNA (Invitrogen). Primer sequences and conditions are available on request.

Paraffin gut tissue sections (normal and HSCR gut, pancreas, cerebral cortex, testis and lung; 5 μm thick) were dewaxed in xylene and rehydrated in a series of graded alcohols. Endogenous peroxidase activity was blocked with water containing 3% H_2_O_2_ for 30 minutes. Antigen retrieval was done by microwaving using citrate phosphate buffer (pH 6.0). Sections were incubated at 4°C overnight with the primary antibodies: IQGAP2 (1:10.000 dilution, mouse monoclonal, Santa Cruz Biotechnology), CHRNA7 (1:5000 dilution, rabbit polyclonal, Sigma Aldrich) and DLC1 (1:2000 dilution, rabbit polyclonal, Sigma Aldrich). After several washes in Tris buffer, peroxidase-labeled secondary antibodies and 3,3'-diaminobenzidine were applied to develop immunoreactivity, according to manufacturer’s protocol (EnVision; Dako, Glostrup, Denmark). The slides were then counterstained with hematoxylin and mounted in DPX (BDH Laboratories, Poole, UK). Sections of cerebral cortex and lung were used as negative controls, while sections of pancreas and testis were used as positive controls to test the three primary antibodies. Photographs were taken at 20 um and 50 um the microscope BX61 (Olympus) with a digital camera DP72 and captured through the CellSens software. Tissues to test the antibodies (all tissues except gut) were selected according the webpage: http://www.proteinatlas.org/.

### Network analysis

The information on protein interactions that define the so called interactome provides an independent view on the biological consistence of the relationship of a set of genes with a disease [41,42]. Here, we have studied the connectivity of the genes discovered in our analysis to the genes already known to be associated to HSCR by means of the program SNOW [43], implemented in the Babelomics package [33]. SNOW detects the largest sub-network linking all these genes and tests if some network parameters are significantly beyond their corresponding random expectations. An extended version of the interactome that includes protein interactions described in Reactome has been used here [44].

An empirical distribution of the random expectation of the parameters of a network of N components can be obtained by repeatedly sampling random sets of N genes from the complete genome and calculating the average parameters of their corresponding minimum connecting trees. Thus, the parameters obtained for the network to be tested can be contrasted with respect to their corresponding random expectations [43].

## Results

### Pathway-based analysis

**Figure 1:**
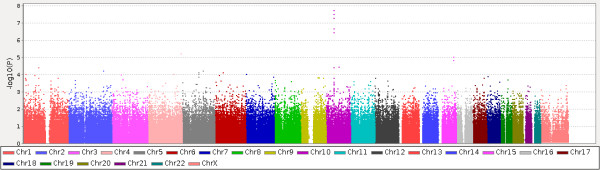
**Graphical summary of the genome wide TDT on the first population of 53 trios used as stage 1 association.** The x axis represents the chromosome position in the consecutive, color-coded chromosomes and the y axis shows -log_10_[p-value]. See details in the text.

The whole workflow-type overview of the steps carried out in this project is reflected in the Additional file 1: Figure S1. Only the *RET* gene was found as significantly associated to HSCR (FDR-adjusted p-value = 7.39x10^-3^) upon the application of a conventional SNP-based TDT on the data resulting from the GWAS in the 53 HSCR trios. Figure 1 contains a graphical representation the distribution of p-values obtained from the TDT.

**Figure 2:**
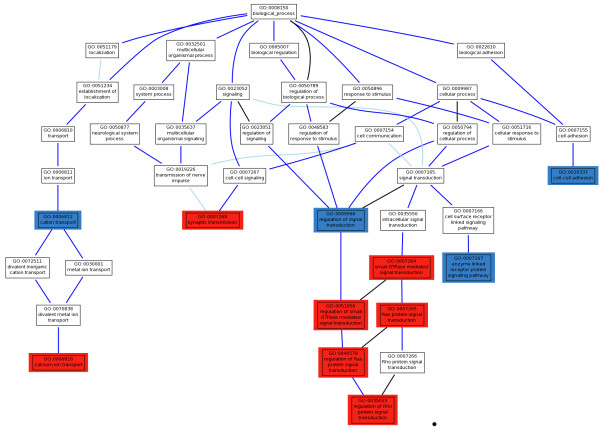
**Hierarchy of GO terms significantly associated to HSCR in the trios analyzed.** GO terms with a highly significant association to the disease (p-value < 10^-3^) to the disease are labeled in red; GO terms with higher, but still significant p-values (between 0.01 and 0.05), that are related to the highly significant GO terms in the GO hierarchy are labeled in blue.

Then, we conducted the PBA test, as implemented in the Babelomics package, over the list of markers ranked by p-value (see Materials and Methods). In this discovery step, we found a total of 11 GO modules significantly associated to HSCR, with FDR-adjusted p-values < 0.01. Five of them were related to signal transduction and its regulation: *Ras protein signal transduction* (GO:0007265), *regulation of Ras protein signal transduction* (GO:0046578), *regulation of small GTPase mediated signal* (GO:0051056), *regulation of Rho protein signal transduction* (GO:0035023) and *small GTPase mediated signal transduction* (GO:0007264). With lower significance, although still significant at FDR-adjusted p-values < 0.05, and belonging to the same GO branch and descendants of the term *signal transduction*, the GO terms *enzyme linked receptor protein signaling pathway* (GO:0007167) and *regulation of signal transduction* (GO:0009966) can also be considered as associated to the disease (see Table 1 and Figure 2). The Ras pathway is a well known intracellular signalling pathway, mediated by the RET receptor and involved in cell survival and proliferation. Both processes are essential in ENS formation [45]. In addition, previous studies have demonstrated that signalling through the small Rho GTPases is also important for colonization of the gut by enteric neural crest cells (ENCC) and the concomitant growth of axons [45]. These results strongly suggest that members of the Ras/Rho protein signal transduction or regulators are most probably playing a key role in the pathogenesis of HSCR, as particularly supported by the association of the GO modules above mentioned, descendant in the GO hierarchy of the *small GTPase mediated signal transduction* term (Figure 2).

**Table 1:** GO modules significantly associated to HSCR (FDR-adjusted p--values < 0.01)

**GO ID**	**Definition**	**Significance**
		**(Adj. p-val < 0.05)**
**GO:0051056**	regulation of small GTPase mediated signal transduction	3.216x10^-5^
**GO:0046578**	regulation of Ras protein signal transduction	6.825x10^-5^
**GO:0007268**	synaptic transmission	4.259 x10^-4^
**GO:0007265**	Ras protein signal transduction	8.245 x10^-4^
**GO:0007264**	small GTPase mediated signal transduction	3.136 x10^-3^
**GO:0035023**	regulation of Rho protein signal transduction	5.369 x10^-3^
**GO:0006816**	calcium ion transport	6.978 x10^-3^
*GO:0016337*	*cell-cell adhesion*	*0.01203*
*GO:0006812*	*cation transport*	*0.02391*
*GO:0007167*	*enzyme linked receptor protein signaling pathway*	*0.03174*
*GO:0009966*	*regulation of signal transduction*	*0.04825*

Values in italics are marginally significant at FDR-adjusted p-values < 0.05.

On the other hand, migration of ENCC in the gut wall during embryogenesis requires interactions between the migrating neural crest cells and the extracellular matrix environment in different regions of the developing gut. Therefore, it results evident the key role of *cell-cell adhesion* (GO:0016337), with a marginal, but still significant (FDR-adjusted p-value < 0.05) association to the disease, during ENS system formation. It is not surprising that genes belonging to this functional module are involved in HSCR. Our analysis also reveals other functional modules associated to the disease, such as *calcium ion transport* (GO:0006816) and, marginally, *cation transport* (GO:0006812), whose biological significance in the context of ENS formation is evidenced by the key role of ionic transport processes in cell migration context. A migrating cell has to govern cell volume regulatory ion transport mechanisms in order to create the appropriate micro- or even nano-environment in the intra- and/or extracellular space, which is necessary to guarantee the correct polarity and hence direction of movement of a migrating cell. Therefore, these associated GOs might provide potential candidate genes to be involved in ENS formation and also the pathogenesis of HSCR.

### Validation of candidate genes

As previously mentioned, a conventional marker-based test rendered only a gene association to the disease, which was, as expected, the *RET* proto-oncogene. Excluding this gene, whose association with the disease has been already extensively reported, we selected a series of 68 additional genes represented in those HSCR-associated GO modules, in order to validate and analyze in more depth the previous functional association results. These 68 genes were those with highest nominal p-values in SNPs mapping onto them. Among those 68 genes, a selection of 190 SNPs was also done (2–3 per gene) and we tested their distribution in the whole series of trios (Additional file 2: Table S1). Genotyping of the selected variants was performed by Taqman technology and their distribution was compared between transmitted and untransmitted alleles from unaffected parents to their affected offspring. This analysis revealed a significant association of 4 out of the 68 genes analyzed, as shown in Table 2. Noteworthy, 2 genes, *DLC1* and *RASGEF1A*, presented association to HSCR with 2 and 3 SNPs respectively, while for the remaining genes only 1 of the tested SNPs resulted in statistical significance. A detailed inspection of the associated genes in the context of the GO modules, showed that 3 of them are part of the signal transduction related terms (*RASGEF1A*, *IQGAP2*, *DLC1*) and the other one is part of GOs related to migration processes (*CHRNA7*) (Table 2).

**Table 2:** Validation of the genes in the stage II, independent population of 106 trios

**Gene**	**SNP**	**Allele**	**Transmited**	**Untransmited**	**Statistic and p-value**
*RASGEF1A (rs1254964 p = 3.856x10*^*-05*^*)*	rs1254958	G	143 (67.45%)	187 (88.2%)	χ ^2^ = 25.27; p = 5x10^-7^
		A	69 (35.55%)	25 (11.8%)	
	rs10793422	G	141 (66.5%)	184 (86.8%)	χ ^2^ = 23.25; p = 1.4 x10^-6^
		A	71 (33.5%)	28 (13.2%)	
	rs2503846	T	32 (15.0%)	71 (33.5%)	χ ^2^ = 18.52; p = 1.68x10^-5^
		G	180 (85.0%)	141 (66.5%)	
*IQGAP2 (rs950643 p = 0.0003585)*	rs3797412	A	146 (68.8%)	179 (84.4%)	χ ^2^ = 13.49; p = 2.393x10^-4^
		G	66 (31.2%)	33 (15.6%)	
*DLC1 (rs1454947 p = 0.007526)*	rs9325866	T	34 (16.0%)	52 (24.5%)	χ ^2^ = 4.22; p = 0.0401
		C	178 (8.04%)	160 (75.5%)	
	rs536147	T	36 (16.9%)	56 (24.5%)	χ^2^ = 5.00; p = 0.0252
		C	176 (83.1%)	156 (75.5%)	
*CHRNA7 (rs2175886 p = 0.000607)*	rs868437	C	47 (22.2%)	76 (35.8%)	χ ^2^ = 8.98; p = 0.0027
		T	165 (77.8%)	136 (64.2%)	

Genes are shown along with the best scoring SNPs mapping onto them and the corresponding p-values obtained in the stage I. See text for details.

**Figure 3:**
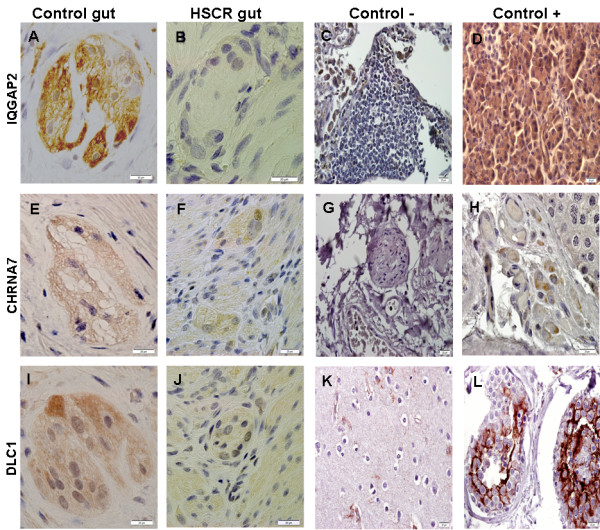
**Expression analysis by immunohistochemistry of IQGAP2 (A-D), CHRNA7 (E-H) and DLC1 (I-L) in human tissues: normal gut (A, E, I), HSCR gut (B, F, J), lung (C, G), pancreas (D), testis (H, L) and cerebral cortex (K).** Both normal and HSCR guts represent myenteric plexuses. Scale bars: 20 μm.

**Figure 4:**
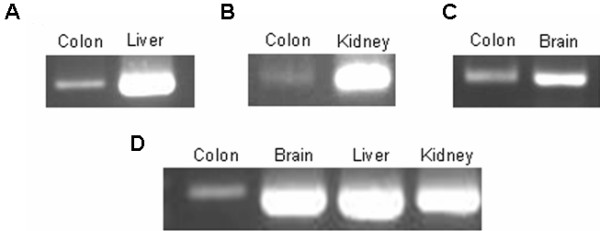
**Expression analysis by RT-PCR of IQGAP2 (A), CHRNA7 (B) and DLC1 (C) in human gut tissues.** cDNAs of kidney, brain and liver have been used as positive controls for RT-PCR. PCR of B-actin was performed with the different cDNAs used in the study (**D**).

In addition, the expression of the 4 genes in human gut specimens was verified by both immunohistochemical (Figure 3) and RT-PCR (Figure 4) analyses.

### Confirmatory network analysis

**Figure 5:**
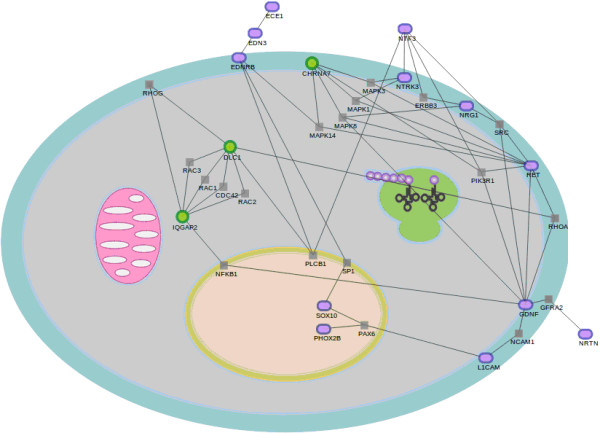
**Sub-network that links disease genes to the new, validated genes found in this work by the application of the program SNOW (see Materials and Methods).** Disease genes are represented as blue ovals, validated genes are represented as green circles and related connecting included by the network analysis procedure are represented with squares.

In order to find additional independent evidences on the biological reliability of the genes validated, we have applied network analysis [41] to look for significant networks that link the discovered genes to already known disease genes. Thus, we applied the network significance test implemented in the SNOW program, which is now part of the Babelomics suite, to the 4 discovered genes and the genes already known to be related to HSCR by numerous studies [1-8]. Figure 5 shows a network that links a group of 12 disease genes and 3 of the 4 validated. This is a considerable number of genes participating in the network even though that there is only information on protein interactions for 11 out of the genes previously related to HSCR, and for 3 out of 4 new genes here identified. This network displays a betweenness value which is significantly higher that the random expectation (p-value = 0.01). This parameter accounts for the number of shortest pathways that connect any two proteins in the sub-network passing through a given node and is an average value. Despite the biological interpretation of network parameters is not always obvious, a high betweenness is characteristic of signaling pathways, which is consistent with the findings based on the GO terms. Both types of evidences point towards processes related to signaling associated to HSCR.

Obviously only genes for which information on the interaction of their gene products is available were used in the derivation of the network. This means that the networks found are most probably an underestimation of the real network associated to the disease.

## Discussion

High-throughput genomic technologies are revolutionizing biology and medicine by providing more and more resolution in the data produced. However, at the same time, are posing new challenges in the way such data can be analyzed and interpreted. In particular, conventional marker-based analysis of GWAS requires of large sample sizes to find significant associations given that some genes may be really associated with the disease, but may not reach a stringent genome-wide significance threshold required in a massive multiple testing scenario [18]. This makes of GWAS a methodology especially impracticable in the field of rare diseases. In order to overcome the limitations of conventional single-marker based association analysis, alternative approaches for the analysis of GWAS analysis have been proposed in the last few years. Beyond testing solutions that use multiple markers or marker information, such as linkage, etc., the recent proposal of PBA approaches has introduced a new angle in the analysis of GWAS data, closer to the principles of systems biology. Typically, PBA approaches check whether some statistics have consistent yet moderate deviation from chance for a group of related genes (for example, belonging to a GO category). The rationale for this approach is the accepted notion that genes do not work in an isolated way, but rather in complex molecular networks and cellular pathways that are often involved in disease susceptibility and progression [23,41,46,47]. Then, the use of prior biological knowledge about relationships among genes and pathways will increase the possibility of identifying the genes and mechanisms that are involved in disease pathogenesis [48]. Here we have adopted a widely accepted definition of gene functionality, which is represented by the GO. One obvious limitation of this approach is that variants not mapping within, or close to, genomic elements with a functional annotation will be missed. However, it is known that coding genes and functional elements in their neighborhood (e.g. splice acceptor and donor sites, transcription factor binding sites, etc.) harbor 85% of the mutations with large disease related effects [49,50].

In this study we have applied a new approach which can be considered a functional version of a two stage analysis of GWAS data. In our approach, the conventional discovery stage based on markers is substituted by a PBA, that allows us to identify biological processes (represented by GO terms) associated to the disease in the Spanish population. The subsequent step of validation allowed us to identify 4 new gene associations to the disease among the ones belonging to the associated GO categories.

It is believed that aganglionosis that characterizes Hirschsprung can arise from: (1) reduced size of the stem/progenitor cell pool; (2) loss of stem cell potency leading to premature differentiation of progenitors; (3) reduced cell survival/ increased cell death; (4) intrinsic, cell-autonomous migration defect of the ENCC; (5) abnormal gut microenvironment; and (6) abnormal interaction of enteric neural crest cells with the gut microenvironment. However, factors that affect the ENCC motility/proliferation/differentiation, and thus probably implicated in HSCR, are not clearly known, as are not how the intrinsic cell migration ability or the NCC-gut microenvironment interaction is controlled at the cellular and molecular level. The PBA here conducted on affected patients, not only confirms the most accepted theory for the biological processes implicated in HSCR, but also provides an important initial hypothesis to select candidate genes for further evaluation in the context of the disease. In this sense, and to reinforce the power of the proposed approach, a significant association to the disease could be confirmed for 4 genes after the evaluation of 68 candidate genes chosen on the basis of their inclusion in the GOs associated to the disease.

Moreover, expression of those genes was demonstrated through RT-PCR and immunohistochemistry analyses in human postnatal gut tissue, obtained from patients with different clinical conditions. Today is widely accepted that neurogenesis takes place in human postnatal ENS, a process which mimics the embryonic events during the formation of the ENS. Due to the limitations of working with human embryonic gut tissue, a postnatal gut context is a powerful tool to identify new genes implicated in the development of ENS, and therefore our results would support again the implication of the tested genes in Hirschsprung disease.

In fact, some of the validated genes had been already related to HSCR either directly or indirectly. That is the case of *RASGEF1A* gene, whose 3 SNPs selected for validation resulted to be all significantly over-transmitted to affected patients. This is not surprising, since *RASGEF1A* is located around 65.5 Kb upstream *RET*, and previous TDT studies had already showed statistically significant disease associations spanning a region immediately 5^′^ of *RET* through to this gene [16] reflecting the high background linkage disequilibrium in this region. *RasGEF1A* acts as very specific guanine nucleotide exchange factor for Rap2, a member of the Rap subfamily of Ras-like G-proteins implicated in the regulation of cell adhesion, the establishment of cell morphology, and the modulation of synapses in neurons [51]. Although association tests have excluded the occurrence of a common mutation at *RASGEF1A* in HSCR, the possibility remains that this gene might carry relevant rare mutations related to HSCR. Importantly, *RASGEF1A* has been reported to be highly expressed in early embryonic development, at a stage coincident with peak *RET* expression and colonization of the gut by neural crest-derived neuronal precursors, which would fit with a potential role in enteric neural crest migration [16]. Regarding *CHRNA7*, it encodes a nicotinic acetylcholine receptor implicated in synaptic transmission regulated by *NRG1* among others [52]. Very interestingly, *NRG1* has been associated to the disease through both common [17,53] and rare variants [6,54]. In addition, *NRG1* SNPs can modulate significantly CHRNA7 expression [52] and for this reason it would be necessary to evaluate the possibility that *NRG1* SNPs associated to HSCR act in combination with *CHRNA7* leading to a susceptibility for the manifestation of HSCR phenotype.

On the other hand, the remaining significant genes have major functions in early embryogenesis or ENS development, which makes conceivable that, although not previously related to HSCR, they might play a role in its pathogenesis. *DLC1* is essential for embryonic development affecting to neural tube development [55] modulating the cytoskeleton and producing morphological changes [56]. *IQGAP2* function is still unclear, although studies in with morpholinos *X. laevis* showed that it regulates cell-cell adhesion during early development [57,58]. According to the network analysis, *IQGAP2* is strongly connected to *DLC1* through five simultaneous connections mediated by the genes *RAC1*, *RAC2*, *RAC3, RHOG* and *CDC42*. It is also connected to *GDNF* (already associated to HSCR) through the *NFKB1* gene. So, despite its still undefined functional role, the experimental evidences of protein interactions firmly link *IQGAP2* to the disease.

In addition valuable information has been also obtained from the network analysis, since it has let us to link the newly identified genes to ones already known to be involved in Hirschsprung. This supports of the associations found and reinforces the role of the new genes in the disease, even though when real network associated to Hirschsprung is probably underestimated because of the lack of information on some gene interactions.

In summary, here we report a number of new candidate genes for HSCR. They need further investigation to elucidate their role in the disease and must be also validated in other populations to discern if their effect in HSCR is universal or is restricted to the Spanish population. Nevertheless, our most important conclusion is that this comprehensive profile of GO terms has demonstrated to be a useful resource for developmental, biochemical and genetic studies. Our report indicate that this approach can help to identify candidate genes for human disease susceptibility loci. These findings could be of especial importance in the field of rare diseases, where large cohorts are often unavailable. In that scenario, the lower sample size requirements make of this approach a suitable and efficient alternative to marker-based analyses.

Beyond technical considerations on the advantages of using GO modules in the analysis of genotype data, the biological pathways highlighted by our study provide insights into the complex nature of HSCR, opens new opportunities for validation of new disease genes and may help in the definition of relatively tractable targets for therapeutic intervention.

### Availability of supporting data

The data sets supporting the results of this article are available in the GEO repository, [http://0-www.ncbi.nlm.nih.gov.opac.acc.msmc.edu/geo/query/acc.cgi?acc=GSE33732].

## Abbreviations

ENCC: Enteric Neural Crest Cells; ENS: Enteric Nervous System; FDR: False Discovery Rate; GO: Gene Ontology; GWAS: Genome-Wide Association Study; HSCR: Hirschprung’s; OMIM: On-line Mendelian Inheritance in Man; PBA: Pathway-Based Analysis; SNP: Single Nucleotide Polymosphism; TCA: Total Colonic Aganglionosis.

## Competing interests

The authors declare that they have no competing interests.

## Authors’ contributions

RMF, SB, GA and JD drafted the manuscript. RMF, RN-T, BL-T, AT and MVE-R carried out the molecular and expression analyses. MB and MM carried out the two-stage analysis. DM supervised the statistical aspects of the work. LG-A carried out the network analysis. IM developed part of the software used in the analysis. JD conceived and coordinated the data analysis. SB conceived the study and coordinated all the laboratory tasks. All authors read and approved the final manuscript.

## Supplementary Material

Additional file 1: Figure S1 Schema of the procedure followed to discover new loci associations. A) The application of a conventional SNP-based TDT on the data resulting from the GWAS in the 53 HSCR trios produces a list of SNPs ranked by p-value. B) SNPs are mapped to genes and genes are thus ranked according to the best (lowest) p-value of the corresponding SNPs. Extragenic SNPs are not used under this approach. C) PBA test produces a list of GO terms significantly over-represented among the genes with best p-values and thus, associated to the disease (see text). D) Genes belonging to the significant GO terms and with a nominal (unadjusted) p-value < 0.05 in the step B are used as First Stage candidate genes. E) New SNPs are selected for these genes and genotyped on an independent cohort. F) The application of an association test produces a list of SNPs that are ranked by FDR-adjusted p-values. G) SNPs with adjusted p-values lower than 0.05 are considered markers for the corresponding loci significantly associated to the disease. H) Network enrichment analysis is conducted to check whether the genes selected are significantly linked among them and to other already known disease genes. Click here for file

Additional file 2: Table S1 Genes included in the study and SNPs selected to evaluate them in our HSCR cohort. Click here for file
