# Supplementary figures and images for: Four new loci associations discovered by pathway-based and network analyses of the genome-wide variability profile of Hirschsprung’s disease
Source: Orphanet J Rare Dis. 2012 Dec 28;7:103. doi: 10.1186/1750-1172-7-103 (PMC3575329; doi:10.1186/1750-1172-7-103)

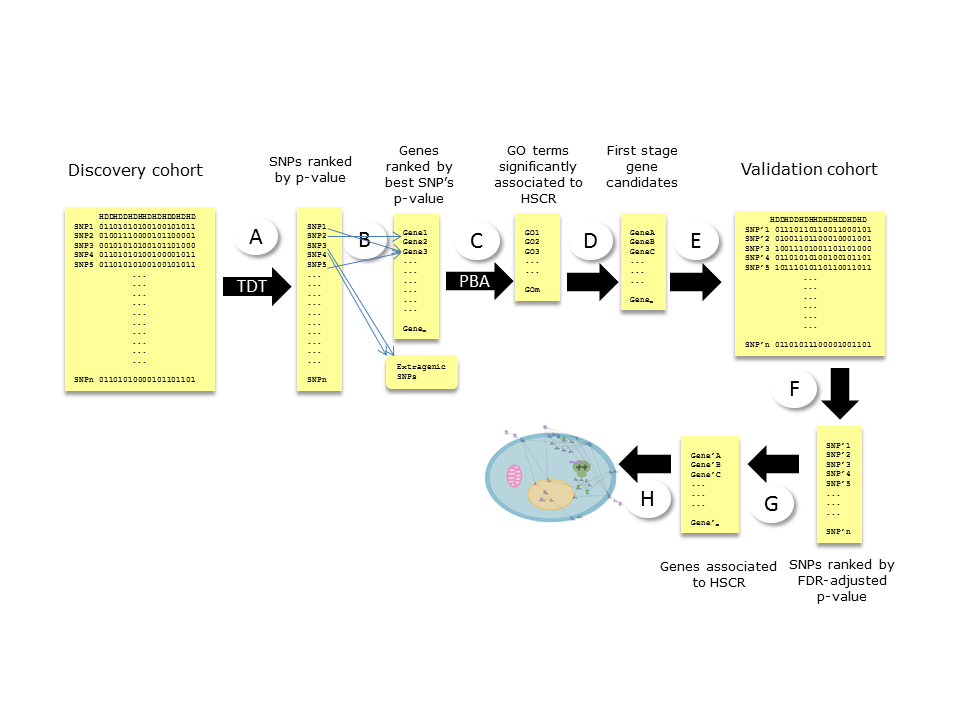

Supplement: Additional file 1: Figure S1 — Schema of the procedure followed to discover new loci associations. A) The application of a conventional SNP-based TDT on the data resulting from the GWAS in the 53 HSCR trios produces a list of SNPs ranked by p-value. B) SNPs are mapped to genes and genes are thus ranked according to the best (lowest) p-value of the corresponding SNPs. Extragenic SNPs are not used under this approach. C) PBA test produces a list of GO terms significantly over-represented among the genes with best p-values and thus, associated to the disease (see text). D) Genes belonging to the significant GO terms and with a nominal (unadjusted) p-value < 0.05 in the step B are used as First Stage candidate genes. E) New SNPs are selected for these genes and genotyped on an independent cohort. F) The application of an association test produces a list of SNPs that are ranked by FDR-adjusted p-values. G) SNPs with adjusted p-values lower than 0.05 are considered markers for the corresponding loci significantly associated to the disease. H) Network enrichment analysis is conducted to check whether the genes selected are significantly linked among them and to other already known disease genes. [file 1750-1172-7-103-S1.tiff]
