# Supplementary material for: Four new loci associations discovered by pathway-based and network analyses of the genome-wide variability profile of Hirschsprung’s disease
Source: Orphanet J Rare Dis. 2012 Dec 28;7:103. doi: 10.1186/1750-1172-7-103 (PMC3575329; doi:10.1186/1750-1172-7-103)
Supplement: Additional file 2: Table S1 — Genes included in the study and SNPs selected to evaluate them in our HSCR cohort. [file 1750-1172-7-103-S2.doc]

| **Gene** | **SNP** |
| --- | --- |
| **AKAP13** | rs11633893 rs17575933 |
| **ARHGEF3** | rs2029466 rs6803697 rs17288908 |
| **BMPR1B** | rs13128703 rs2510540 |
| **CACNA1C** | rs758723 rs2283326 rs4765960 |
| **CACNA2D3** | rs9827201 rs7652209 rs10510770 |
| **CARD11** | rs11773360 rs7789581 rs7806365 |
| **CDH13** | rs12597394 rs427585 rs7184056 |
| **CDH2** | rs12519679 rs11951124 rs4701563 |
| **CDH23** | rs7094749 rs1234888 rs1227079 |
| **CDH4** | rs6124086 rs2236070 |
| **CEACAM21** | rs2302188 rs1076362 rs3745936 |
| **CHRDL1** | rs197036 rs197023 |
| **CHRNA1** | rs3816448 rs2600685 rs12997022 |
| **CHRNA7** | rs7179008 rs2611605 rs868437 |
| **CLSTN2** | rs4361242 rs4271881 rs9876855 |
| **CNTN4** | rs4685593 rs6442761 rs6442762 |
| **COL13A1** | rs4746014 rs2683555 rs2704495 |
| **CTNNA3** | rs10733829 rs1911326 rs10822705 |
| **CTNND2** | rs6873547 rs852640 rs6880938 |
| **DLC1** | rs2044121 rs9325866 rs836147 |
| **DLGAP1** | rs1791378 rs11081062 rs582324 |
| **EGFR** | rs11773818 rs759162 rs 17172451 |
| **EPHA3** | rs9875278 rs11916046 rs7639401 |
| **ERCC5** | rs751402 rs7325708 rs4150355 |
| **FARP2** | rs10172825 rs1476698 rs2055568 |
| **FAT3** | rs11606920 rs6439695 rs1488859 |
| **FN1** | rs1250241 rs17517928 rs13423742 |
| **GABRB1** | rs6447541 rs6819531 rs959160 |
| **GABBR2** | rs570138 rs1571929 rs2779543 |
| **GABRR1** | rs450597 rs13215029 rs11756847 |
| **GLRA3** | rs10520290 rs9998745 rs12507854 |
| **GPR98** | rs10514343 rs2063245 rs2697548 |
| **GRM7** | rs3749448 rs779864 rs3804886 |
| **HTR1E** | rs1408449 rs942472 rs828358 |
| **IQGAP2** | rs3797412 rs10056943 rs10038589 |
| **KCNIP1** | rs1363709 rs9313507 rs329476 |
| **KCNQ5** | rs12201380 rs10943055 rs4707978 |
| **KCNMA1** | rs620803 rs1356092 |
| **MBP** | rs470797 rs470443 rs9676113 |
| **MCM7** | rs2070215 |
| **NCAM2** | rs2826815 rs2826808 rs1017739 |
| **NLGN1** | rs6777101 rs1549100 rs1553122 |
| **NRXN3** | rs221514 rs17764956 rs1424842 |
| **OPCLM** | rs3019866 rs1939966 rs2246352 |
| **OTOG** | rs11024345 rs7107432 rs10832811 |
| **PAX3** | rs10932949 rs10932951 rs1978859 |
| **PHLDB2** | rs13077 rs16858936 rs2137897 |
| **PIK3R1** | rs251409 rs12697060 rs16897558 |
| **PKHD1** | rs9370079 rs1266924 rs927185 |
| **PREX2** | rs6980865 rs17445916 rs6998813 |
| **PRKCA** | rs12938937 rs10853078 rs12600582 |
| **PTPRT** | rs17810209 rs17314843 rs6065524 |
| **RABGAP1L** | rs333422 rs2073798 |
| **RASGEF1A** | rs1254958 rs10793422 rs2503846 |
| **RDX** | rs10789756 rs11605530 rs11606391 |
| **RGS6** | rs2283385 rs11158942 rs12883063 |
| **ROBO2** | rs12492221 rs4432692 rs9859970 |
| **RYR3** | rs1435118 rs4780153 |
| **SIPA1L3** | rs705495 rs833904 rs833915 |
| **SLC16A7** | rs12231740 rs10877333 rs3847656 |
| **SYN2** | rs521223 rs3773364 rs422584 |
| **TGFA** | rs10172814 rs375668 rs446086 |
| **TIAM1** | rs2268231 rs2833308 rs2248656 |
| **TNFRSF11A** | rs8083511 rs8092023 rs12970081 |
| **VAV3** | rs4514263 rs17477236 |
| **VDAC1** | rs4958205 rs3733946 rs11952145 |
| **ZAN** | rs6942733 |
| **ZCWPW1** | rs6465770 |

Supplementary Table 1. Genes included in the study and SNPs selected to evaluate them in our HSCR cohort.
